# Vitality of Infants

**Published:** 1881-11

**Authors:** 


### VITALITY OF INFANTS.

Some statistics on this subject have recently been published by Baron Kolb of Germany. Out of 100 children nursed by their mothers, only 18.2 died during the first year; of those nursed by wet-nurses, 29.33 died; of those artificially fed, 60 died; of those brought up in institutions, 80 died in the 100. Taking 1000 well-to-do persons and another 1000 of poor persons, after five years there remained of the prosperous, 943; of the poor only 655. After fifty years there remained of the prosperous 557; of the poor, 283; at seventy years of age there remained 235 of the prosperous, and of the poor only 65. The average length of life among the well-to-do was fifty years, among the poor, thirty-two years.

These comparative figures are very greatly at variance with general impressions. Most persons are impressed with the idea that the poor are, as a general thing, longer-lived than the wealthy, but Baron Kolb, if his statistics are reliable, and it is claimed they are, wholly overturns that belief. The author furnishes some reasons for the marked difference in point of vitality of the two classes, prominent among which is "the anxiety of providing for bare subsistence, and the lack of proper sanitary conditions, as well as the lack of abundance of such food and clothing as are needed to sustain the body properly.

